# Chronic Inflammatory Demyelinating Polyneuropathy and Evaluation of the Visual Evoked Potentials: A Review of the Literature

**DOI:** 10.3390/medicina59122160

**Published:** 2023-12-13

**Authors:** Periklis Tsoumanis, Aikaterini Kitsouli, Christos Stefanou, Georgios Papathanakos, Stefanos Stefanou, Kostas Tepelenis, Hercules Zikidis, Afroditi Tsoumani, Paraskevas Zafeiropoulos, Panagiotis Kitsoulis, Panagiotis Kanavaros

**Affiliations:** 1 Department of Ophthalmology, University Hospital of Ioannina, 45500 Ioannina, Greece; pariszafeirop@gmail.com; 2 Anatomy-Histology-Embryology, University of Ioannina, 45500 Ioannina, Greece; katerinakitsouli@gmail.com (A.K.); pkitsoulis@hotmail.com (P.K.); pkanavar@uoi.gr (P.K.); 3 Department of Surgery, General Hospital of Filiates, 46300 Filiates, Greece; christosstefanou.gr@gmail.com; 4 Intensive Care Unit, University Hospital of Ioannina, 45500 Ioannina, Greece; gppthan@icloud.com; 5 Department of Endocrine Surgery, Henry Dunant Hospital Center, 11526 Athens, Greece; stefanos23s@gmail.com; 6 Department of Surgery, General Hospital of Ioannina G. Hatzikosta, 45500 Ioannina, Greece; kostastepelenis@gmail.com; 7 Department of Neurology, University Hospital of Ioannina, 45500 Ioannina, Greece; herczkd@gmail.com; 8 Department of Neurology, G. Hatzikosta, 45500 Ioannina, Greece; aftsoumani@gmail.com

**Keywords:** CIDP, epidemiology, etiology, atypical manifestations, treatment, visual evoked potentials

## Abstract

Chronic inflammatory demyelinating polyneuropathy (CIDP) is a rare autoimmune disorder characterised by the progressive demyelination of peripheral nerves, resulting in motor and sensory deficits. While much research has focused on clinical and electrophysiological aspects of CIDP, there is an emerging interest in exploring its impact on the visual system through visual evoked potentials (VEPs). This comprehensive review synthesises existing literature on VEP findings in CIDP patients, shedding light on their potential diagnostic and prognostic value. The review thoroughly examines studies spanning the last two decades, exploring VEP abnormalities in CIDP patients. Notably, VEP studies have consistently revealed prolonged latencies and reduced amplitudes in CIDP patients compared to healthy controls. These alterations in VEP parameters suggest that the demyelinating process extends beyond the peripheral nervous system to affect the central nervous system, particularly the optic nerve and its connections. The correlation between VEP abnormalities and clinical manifestations of CIDP, such as visual impairment and sensory deficits, underscores the clinical relevance of VEP assessment in CIDP management. Furthermore, this review addresses the potential utility of VEPs in aiding CIDP diagnosis and monitoring disease progression. VEP abnormalities may serve as valuable biomarkers for disease activity, helping clinicians make timely therapeutic decisions. Moreover, this review discusses the limitations and challenges associated with VEP assessment in CIDP, including variability in recording techniques and the need for standardised protocols. In conclusion, this review highlights the evolving role of VEPs as a non-invasive tool in CIDP evaluation. The consistent VEP abnormalities observed in CIDP patients suggest the involvement of the central nervous system in this demyelinating disorder. As our understanding of CIDP and its pathophysiology continues to evolve, further research in this area may lead to improved diagnostic accuracy and monitoring strategies, ultimately enhancing the clinical management of CIDP patients.

## 1. Introduction

Chronic inflammatory demyelinating polyneuropathy (CIDP) is an autoimmune illness portrayed by progressive peripheral neuropathy with antibodies turning against peripheral nerve myelin [1]. It contains several subtypes, which, in most cases, are treatable neuropathies. The European Federation of Neurological Societies (EFNS) and the Peripheral Nerve Society (PNS) describe the disease as progressive, that it has demyelination symptoms and responds positively to immune-modulating treatments [2]. Surveys propose that physical activity might enhance motor disorders in patients with CIDP. The exercise was related to “lower sensory impairment” [3].

The disease can be either typical or atypical. The typical form of disease starts with tingling sensations and weakness in the furthest parts of the limbs, along with challenges in walking. A clinical examination reveals a gradual weakening of muscles near and far from the body’s centre, loss of sensation, and reduced or absent reflexes in deep tendons. This condition tends to worsen for over eight, but might also have periods of improvement followed by relapses [4]. This specific form of chronic inflammatory demyelinating polyneuropathy (CIDP) is more prevalent in males. It can occur at any age, commonly between 40 and 60, but it is also possible during infancy and childhood [5].

Scientists have attempted to comprehend if a visual pathway is ruined in patients with the typical form of CIDP [1]. Hawke et al. mentioned ‘high white matter signals’ in patients’ MRIs [6].

Half a century ago, Austin described CIDP for the first time. CIDP may be challenging, but an early diagnosis is significant to anticipating permanent nerve damage. The typical form is exhibited by different clinical views [7]. It might affect children, although this phenomenon is rare. Diagnosis is demanding, yet an immediate identification has positive outcomes in the treatment. Regarding the differential diagnosis, the illness’ course modification and new signs should be considered [8].

Clinical presentations different from typical CIDP are considered CIDP variants (Table 1) because they share the standard features of demyelination and response to immune therapy. These variants are the following:

- Distal acquired demyelinating symmetric neuropathy (DADS),
- Motor CIDP,
- Sensory CIDP,
- Multifocal CIDP,
- Focal CIDP.

Distal CIDP, or distal acquired demyelinating symmetric neuropathy [9], manifests as a loss of sensation in the outer parts of the upper and lower limbs alongside difficulty walking steadily. Weakness might occur, usually more pronounced in the outer regions of the lower limbs than the upper ones. About two-thirds of individuals displaying this characteristic have IgM paraprotein neuropathy, often with antibodies targeting myelin-associated glycoprotein (MAG) [10,11]. A distal neuropathy accompanied by an IgM paraprotein and anti-MAG antibodies, known as anti-MAG neuropathy, is considered distinct from CIDP. Most patients with this condition exhibit specific electrodiagnostic and pathological findings and do not show positive responses to intravenous immunoglobulin (IVIg) or corticosteroids.

Multifocal CIDP, or multifocal demyelinating neuropathy with persistent conduction block, Lewis-Sumner syndrome (LSS), multifocal acquired demyelinating sensory and motor neuropathy (MADSAM), or multifocal inflammatory demyelinating neuropathy [12], typically starts by impacting the upper limbs. Eventually, the lower limbs might also be affected, either later during the condition or sometimes right from the beginning. In this form, specific cranial nerves, such as the oculomotor, trigeminal, facial, vagal, and hypoglossal nerves, are likely to be more frequently affected than other CIDP types [13].

Focal CIDP is uncommon and typically impacts the brachial or lumbosacral plexus, although it can also involve specific peripheral nerves [14].

Motor CIDP appears as a balanced weakening in both the inner and outer parts of the limbs without any clinical or electrodiagnostic abnormalities in sensation [15]. This differs from typical CIDP, where sensory irregularities and multifocal motor neuropathy (MMN) display an uneven weakening pattern primarily affecting the upper limbs [16]. When sensory nerve conduction shows irregularities in clinically diagnosed motor CIDP, it is termed motor-predominant CIDP. Patients with motor CIDP might experience worsening conditions even after corticosteroid treatments [17,18].

Sensory CIDP is commonly identified by unsteady walking, reduced ability to sense vibrations and positions, and alterations in skin sensation [19]. Notably, muscle weakness is not a defining characteristic. When there’s a presence of slowed motor nerve conduction or a block in motor conduction, it is termed sensory-predominant CIDP.

## 2. Methodology

### 2.1. Literature Search Strategy

We conducted a systematic search of peer-reviewed literature using electronic databases, including PubMed, Scopus, and Web of Science. The search was performed from January 1990 to December 2021 to capture the most recent studies related to chronic inflammatory demyelinating polyneuropathy (CIDP).

### 2.2. Inclusion and Exclusion Criteria

Studies were included if they met the following criteria:

- peer-reviewed articles published in English,
- focused on chronic inflammatory demyelinating polyneuropathy (CIDP) and
- presented original research findings or comprehensive reviews.

Studies were excluded if they were:

- conference abstracts,
- not accessible in full-text, or
- did not pertain to the scope of this review.

## 3. Limitations

This review aims to provide a comprehensive overview of the current literature concerning the diagnosis, treatment modalities, and prognosis of chronic inflammatory demyelinating polyneuropathy (CIDP). However, certain limitations in the scope should be acknowledged.

Primarily, the review focused on articles published in peer-reviewed journals from 1990 to 2021, predominantly available in English. While efforts were made to encompass a wide range of research, restricting the search to this time frame and language might have excluded valuable studies published earlier or in languages other than English, potentially limiting the inclusivity of findings.

Furthermore, the review primarily concentrated on clinical aspects, including diagnostic criteria, therapeutic interventions, and prognostic factors.

Additionally, this review mainly addressed CIDP in the adult population. Studies primarily focused on paediatric CIDP cases were not extensively covered due to the specific focus on adult populations.

Lastly, while efforts were made to include various treatment modalities (intravenous immunoglobulin, corticosteroids, etc.), the review might not encompass all emerging or experimental therapies due to the evolving nature of CIDP treatment approaches.

## 4. Pathology

A blend of autopsy, MRI, and ultrasound examinations has revealed that inflammatory lesions in CIDP (chronic inflammatory demyelinating polyneuropathy) primarily manifest in the spinal roots, proximal nerve trunks, and significant nerve networks, although they can also appear dispersed throughout the peripheral nervous system (PNS) [20]. However, because accessing proximal nerves and nerve roots is relatively challenging, most biopsies are extracted from the sural nerve. Despite this site being distant from the most noticeable inflammatory activity, pathological alterations in sural nerve biopsies encompass a wide range of changes. These changes span from no abnormalities to manifestations such as swelling, demyelination, the formation of onion-like structures (onion bulbs), axonal degradation, and inflammatory infiltrates of macrophages [21] and T cells around blood vessels or within nerve tissue. These pathological variations are also observable in an animal model of CIDP called experimental autoimmune neuritis (EAN), which is induced in susceptible rodent or rabbit strains through immunization with either complete myelin or specific myelin proteins. EAN mirrors an autoimmune assault on peripheral nerves orchestrated by the immune response’s cellular and humoral components.

## 5. Etiology

CIDP is a progressive immune-mediated peripheral neuropathy where T-cells and humoral immune mechanisms revolve around the peripheral nerve myelin. The etiology of chronic inflammatory demyelinating polyneuropathy (CIDP) is multifactorial, involving a complex interplay of genetic susceptibility, autoimmune mechanisms, and environmental factors. While the exact cause remains elusive, it is widely believed that CIDP results from an autoimmune response targeting the peripheral nervous system. This immune dysregulation is thought to be triggered by various factors, including viral infections, such as the Epstein–Barr virus and the cytomegalovirus, which can initiate an aberrant immune response leading to demyelination [22,23]. Genetic predisposition may also play a role, as specific HLA genotypes have been associated with an increased risk of CIDP [24]. Furthermore, the production of autoantibodies against specific nerve components, such as myelin-associated glycoprotein (MAG) or gangliosides, is implicated in the pathogenesis of CIDP [25]. The exact interplay of these factors in the development of CIDP remains an active area of research, and a comprehensive understanding of its etiology is critical for the development of targeted therapies and improved patient care.

## 6. Epidemiology

Even though CIDP is the most familiar chronic neuropathy, its prevalence is low. Chronic inflammatory demyelinating polyneuropathy (CIDP) is a rare neurological disorder, and its epidemiology varies across different regions. CIDP is estimated to have an annual incidence of 0.4 to 3.6 per 100,000 individuals and a prevalence ranging from 1.9 to 7.7 per 100,000 people [26]. The condition affects both genders, with a slight male predominance, and it often manifests in mid to late adulthood, although it can occur in individuals of all ages [27,28]. Another example is Minnesota, where, in 2000, the average age of patients was 58 years. The average illness period was ten months, consisting of men over women. Furthermore, the clinical presentation of CIDP can vary widely, making diagnosis and epidemiological assessments challenging. These statistics highlight the rarity of CIDP and emphasise the need for ongoing research to understand better its epidemiology, risk factors, and geographic variations.

## 7. Clinical Presentation

CIDP is a heterogeneous neurological disorder characterised by a broad spectrum of clinical presentations, making diagnosis and management challenging. Patients with CIDP may exhibit various clinical features, and the presentation can range from mild, sensory-only forms to severe, disabling motor and sensory involvement.

Typically, CIDP presents with a subacute or chronic onset and progresses over an extended period. The hallmark of CIDP is a symmetric and predominantly motor involvement with sensory disturbances. Standard clinical features include progressive limb weakness, loss of deep tendon reflexes, sensory deficits (numbness, tingling, and paresthesias), and sensory ataxia [29]. These symptoms often ascend from the distal limbs to proximal muscles, a characteristic pattern known as the “dying-back” phenomenon.

Furthermore, CIDP can manifest in various clinical phenotypes, such as the classic form, multifocal acquired demyelinating sensory and motor neuropathy (MADSAM), distal acquired demyelinating symmetric (DADS) neuropathy, and pure sensory CIDP [30]. These clinical subtypes may have specific features, including variable cranial nerve involvement, upper limb or lower limb predominance, and predominantly sensory or motor impairment.

CIDP may also present atypical features, including pain, fatigue, autonomic dysfunction, or cranial nerve involvement. Moreover, some patients may experience relapsing-remitting courses, further complicating the clinical picture [16].

In conclusion, CIDP is a clinically heterogeneous disorder with a wide array of presentations, making it essential for clinicians to be vigilant and consider CIDP in the differential diagnosis of patients with progressive motor and sensory deficits. Early recognition of CIDP and applying diagnostic criteria are crucial for appropriate management and therapeutic interventions.

## 8. Diagnosis

Diagnosing chronic inflammatory demyelinating polyneuropathy (CIDP) can be a complex process due to its varied clinical presentation. However, several diagnostic criteria and tests are commonly used to establish a CIDP diagnosis.

(a) Clinical Evaluation: CIDP often presents with a progressive or relapsing symmetric weakness affecting the limbs, alongside sensory disturbances. Clinically, it is essential to exclude other potential causes of neuropathy. The examination should also consider cranial nerve involvement, autonomic dysfunction, and atypical features [16].
(b) Electrophysiological Studies: Nerve conduction studies (NCS) are pivotal in diagnosing CIDP. Characteristic NCS findings include evidence of demyelination, such as slowed nerve conduction velocities, prolonged distal latencies, conduction block, and temporal dispersion [16]. According to the 2010 EFNS/PNS guidelines [16], Van den Bergh et al. [4] recommended using nerve conduction studies (electrodiagnosis) and clinical identification to diagnose typical CIDP and its variants. They simplified the levels of certainty in electrodiagnostic assessments to just two: CIDP and possible CIDP, as electrodiagnostic criteria for probable and definite CIDP have similar sensitivity and specificity [31]. As no gold standard for CIDP diagnosis exists, the task force recommended not using the “definite CIDP”. Including sensory and motor studies is mandated to define the diagnostic classifications for typical CIDP and its variants.
(c) Cerebrospinal Fluid (CSF) Analysis: In some cases, an elevated CSF protein concentration without an increase in white blood cells (albuminocytologic dissociation) can support the diagnosis of CIDP.
(d) Immunological and Serological Tests: Certain antibodies, such as anti-ganglioside antibodies or anti-myelin-associated glycoprotein (MAG) antibodies, may be present in some CIDP patients, aiding in diagnosis [32]. Besides, it is found that there is a relation between “anti-NF 155 IgG4 antibodies” and CIDP in a study; 5% of patients (three of 55) were found with anti-NF155 IgG, and these autoantibodies seem to be a “biomarker” to enhance patients’ diagnosis and therapy. Patients with more significant symptoms had more autoantibodies [33].
  Immunological and serological tests are essential components used in diagnosing CIDP and its variants, often aiding in confirming the condition or ruling out other potential causes. These tests include assessments for various antibodies and markers in the blood or cerebrospinal fluid, helping to elucidate the immune system’s involvement in CIDP. It is important to note that while these tests are integral in evaluating CIDP, nodopathies, which involve disorders characterised by abnormalities at the site of the nerve nodes, are currently not considered part of the CIDP diagnostic framework [34]. Diagnosis of CIDP is primarily centred around demyelination or damage occurring in the peripheral nerves rather than specific nodal abnormalities. Therefore, nodopathies are not typically associated with or categorised within the spectrum of CIDP diagnosis.
(e) Magnetic Resonance Imaging (MRI): MRI of the brachial and lumbosacral plexus may reveal hypertrophy or contrast enhancement, which can support the diagnosis [35]. Measurements of the peripheral nerve volume and scan techniques such as magnetic resonance (MR) neurography” and diffusion tensor imaging (DTI) are significant for the diagnosis and the evaluation of CIDP [36]. DTI amounts in CIDP are significantly lower in the nerves of all four limbs, and T2 hyperintensity is noticed both in the typical and atypical forms of CIDP. Kronlange et al. [37] state that an evolved MRI called “three-dimensional nerve sheath signal increased with inked-reduced tissue rapid acquisition of relaxation imaging” illustrates increased ganglia and roots in patients approximately twice the size of healthy people.
  Ideally, a quantitative evaluation of the sizes of spinal nerve roots is preferred, which involves measuring the nerve root diameter adjacent to the ganglion in the coronal plane. The criterion for abnormality is a measurement exceeding 5 mm in height. Alternatively, a semi-quantitative method involves categorizing abnormalities of the spinal nerve roots and trunks into three groups: normal, potentially abnormal, or distinctly abnormal [38].
(f) Ultrasound: In most cases, ultrasound portrays “enlarged cross-sectional areas (CSA) in impacted nerves”, but the results can upsize the diagnosis for CIDP compared with other neuropathies. Ultrasound findings of these patients include nerve magnification [39]. An ultrasound provides a safer diagnosis by segregating diabetic demyelinating sensorimotor neuropathy and CIDP.
  CIDP diagnosis might lean towards likelihood when there is an observed nerve enlargement in at least two areas within the proximal median nerve segments or the brachial plexus. This includes a median nerve cross-sectional area exceeding specific measurements: >10 mm^2^ in the forearm, >13 mm^2^ in the upper arm, >9 mm^2^ in the inter scalene (trunks), or >12 mm^2^ for nerve roots. It is important to note that no current evidence supports using ultrasound for diagnosing CIDP in paediatric patients [4].
(g) Nerve Biopsy (rarely used): Nerve biopsy may be considered when other diagnostic methods are inconclusive. When CIDP is suspected, but treatment yields minimal or no response, prompting consideration of alternative diagnoses like CMT, amyloidosis, sarcoidosis, or nerve sheath tumours/neurofibromatosis. The sural or superficial peroneal nerve is commonly chosen when conducting a nerve biopsy. However, biopsying a nerve that is clinically affected is more likely to yield valuable information. Typical histopathological features include demyelination and inflammatory infiltrates in nerve fascicles [40], thinly myelinated axons and small onion bulbs, thinly myelinated or demyelinated internodes in teased fibres, perivascular macrophage clusters and supportive features of demyelination on electron microscopy.

It is crucial to apply a combination of these diagnostic approaches to confirm a CIDP diagnosis, as the disorder’s presentation can be variable. The criteria proposed by the European Federation of Neurological Societies (EFNS) and Peripheral Nerve Society (PNS) provides a valuable framework to standardise CIDP diagnosis, ensuring patients receive accurate and timely identification and treatment.

## 9. Clinical Utility of Visual Evoked Potentials in Diagnosing CIDP

The assessment of visual evoked potentials (VEPs) in chronic inflammatory demyelinating polyneuropathy (CIDP) serves as a valuable diagnostic tool to evaluate potential central nervous system (CNS) involvement in a primarily peripheral nerve disorder. CIDP is traditionally characterised by demyelination of the peripheral nerves, but in some cases, patients may exhibit subclinical or overt CNS demyelination. VEPs, which measure the electrical responses generated in the brain’s visual cortex in response to visual stimuli, can reveal prolonged latencies and bilateral asymmetry in CIDP patients with CNS involvement. This diagnostic approach is beneficial in atypical cases or when clinical symptoms and other criteria suggest the possibility of CNS demyelination. Regular VEP assessments can also aid in monitoring disease progression and evaluating treatment responses in CIDP patients with CNS involvement. While not all CIDP patients require VEP testing, it is a valuable adjunct in cases where CNS involvement is suspected or atypical presentations of the disease, contributing to a more comprehensive understanding of the condition.

It is found that patients with CIDP might have abnormalities in visual evoked potentials (VEPs) when compared with the healthy population. VEP is an electrodiagnostic test that records glial cells’ responses to the S system subcortical visual path and brain neurons around the occipital lobe. For this test, dispersed bright stimuli are used, and some contact electrons are placed on the skin of the head.

Differences between the healthy population and the patients are found in the diagnostic markers P100, N145, and N175 [1]. These results lead us to conclude that there are data differences in the range of VEP numbers between the two teams. Authors Dziadkowiak et al. [1] conducted a case-control study of two groups to assess VEPs in patients with CIDP. The first group consisted of 24 patients (16 male, 8 female) with an average age of 60 years who satisfied the requirements for CIDP by the European Federation of Neurological Societies/Peripheral Nerve Society Instructions [16]. In total, 13 people among 24 had CIDP not more than 6 months, 6 of 24 patients had CIDP for one year, and 5 of 24 had CIDP between 1 and 3 years. The second control group comprises 35 healthy people whose age and gender were compatible. The guidance material of the International Federation of Clinical Neurophysiology examined the VEPs. They were provoked by “structural chess stimulus”, switching white and black sectors using a Nicolette monitor at 1 m. space. N75, P100, and N145 variate latencies and the dispute in P100 latency and P100-N145 width were evaluated. All the participants were probably qualified for CIDP. The duration of clinical indicators was one year. In total, 13 patients had latency in VEPs’ parameters. In the first group, latencies of N75 and P100 were more significant in length than in the second group [41].

Hawke et al. [6] mentioned irregular MRI scans portraying high white signals in 6 of 26 patients. Previous surveys propose the CNS fusion, containing the visual pathway, which can also happen in CIDP. Furthermore, 14 of 28 patients presented a raw of 6 incidents. Five of these six incidents revealed prolonged VEPs latencies and T2 signals on MRI scans. Also, in a collocated central and peripheral demyelination survey, 15 of 21 patients revealed atypical VEPs. A case-control study was conducted with patients’ total age and sex-matched with the controls. All these people were negative for ocular diseases and other pathologies. This report aims to research whether VEPs examination is appropriate to trace oculus disorders in patients with CIDP devoid of symptoms. In this research [42], the standards were the adulthood, possible or confirmed CIDP by CIDP Guidelines EFNS/PNS and their correspondence to corticosteroid therapy. Team A comprised 43 patients, and Team B comprised 18 healthy people. From the first team, 18 people participated in the study, and 25 were excluded due to ophthalmic diseases and other pathologies. The final form of these groups was 18 patients with CIDP and 18 healthy controls. The outcomes were the following. In group A, VEP examination showed a width of 182.06 + 437 ms and latency of 149.65 + 6.51 ms. Group B analysis revealed a width of 183.53 + 43.85 ms and a latency of 147.84 + 5.68 ms. There were no significant variations in widths between the two groups. These results cannot exclude disparities between the two groups due to the small sample number [42].

A survey analyses the visual evoked potentials in 10 people diagnosed with CIDP compared to the controls—25 people to cross probable signs of CNS demyelination—VEPs examination achieved in every person with CIDP 1 month before and after medication with IVIG. Patients were chosen according to Joint Task, EFNS and PNS gaudiness [16]. The age’s width was between 38 and 77 years. The amplitude of disease in patients was between 4 months and ten years. All people with a particular illness underwent an ophthalmological examination to exclude other ophthalmic pathologies influencing VEP results. VEP examination achieved before 2 and 4 weeks after medication with IVIG. In controls, P100 latency was 102.5 + 9.2 ms and 7.2 + 2.8 ms. The range between 120.9 ms and fewer than 1.8 ms is regarded as aberrant.

The outcomes for P100 latency and the range of VEPs for ten patients are summed up. In comparison with healthy subjects, three patients had boosted P100 latency, which was lower after the IVIG medication and patients had no motor weakness. P100 latency was in ordinary amplitude in seven patients and stayed this way after receiving IVIG treatment [43].

## 10. Treatment

Initial treatment of CIDP with the most effective medications is significant to anticipate other deteriorations. Treatment for CIDP is separated into initial and maintenance therapy. Plasma exchange, corticosteroids, and intravenous immunoglobulin are recognised as efficient therapies. Nonetheless, hematopoietic stem cell transplantation (HSCT), used to cure various autoimmune diseases, might also have a therapeutic implication on CIDP [44]. However, HSCT and chemotherapy are mainly used only for refractory CIDP cases.

Methotrexate was ineffective in a survey where intravenous immunoglobulin or corticosteroids were administrated as maintenance therapy [45]. The usual dose of intravenous immunoglobulin is 2 g/kg bodyweight for 2–5 days. Some patients require one or two sessions to show improvement, while others need more than two sessions to upgrade. The presence of more significant vascular risk indicators in patients who receive long-term IVIg as treatment should be mentioned more than other people [46]. Intravenous immunoglobulin expands short-term disabilities and is better than previous therapies [47], while subcutaneous immunoglobulin can also constitute therapy in the initial form of illness.

Short-term therapy with subcutaneous immunoglobulin (SCIG) and intravenous immunoglobulin (IVIG) showed the same grade’s enhanced motor presentation; however, IVIG patients were enhanced earlier than SCIG therapy [48]. In their study, Markvardsen et al. [48] proved that patients with CIDP can still upgrade to another treatment when the first choice is inappropriate [49]. In a survey monitoring the administration of two doses of immunoglobulins SCIG and IgPro2020, experts concluded that both were efficient as maintenance therapies for CIDP [50]. Human 20% liquid, known as IgPro20, is the most common SCIg, authorised in the USA and EU as a maintenance treatment [51]. Maintenance therapy with IVIG consists of efficient treatment for patients with CIDP, but the administration happens every three weeks on the prerequisite. These patients should be monitored for “thrombotic complications”, especially in people with vascular disorders [32]. SCIG is a secure and effective therapy, switching to IVIG and providing the possibility of administration at home, minimising the risk of exposure to SARS-CoV-2. The alternating from IVIG to SCIG therapy should be adapted to each individual, based on each patient’s needs. However, during the coronavirus pandemic, it should be reflected to decrease the prevalence of COVID-19 [52].

Some patients show better results if treatment begins with corticosteroids (prednisolone per os, high-dose dexamethasone or intravenous methylprednisolone), but there are no indications about the most efficient corticosteroid therapy. It is identified that 60% of patients respond to corticosteroid therapy and respite in 61% of therapy “responders” [53]. Worth noting is that long-term administration with corticosteroids has severe side effects. “Pulsed intravenous corticosteroids” have fewer severe side effects than regular daily use [54]. “Pulse oral corticosteroid” treatment has better results than switching therapy to intravenous IVIg [55].

In patients with CIDP who do not reciprocate to intravenous immunoglobulin or corticosteroids and in patients with severe symptoms, plasma exchange can be considered as an alternative. Although rare, plasma exchange seems promising for pregnant women [56]. Moreover, intravenous immunoglobulin has shown more immediate improvement than noticed with corticosteroids.

The side effects of following intravenous immunoglobulin therapy are less in comparison with corticosteroids. A cohort survey revealed the following results: 76% of patients reciprocated to intravenous immunoglobulin therapy, and the 24% who did not respond to intravenous immunoglobulin were submitted to treatment with plasma exchange or corticosteroids.

In a case-report survey, scientists proved that in some subtypes of CIDP, bortezomib could be an efficient way of treating CIDP [57]. Rituximab is also an effective treatment for patients with haematological and other auto-immune disorders [58].

These diseases can be completely recovered but remain unacknowledged [59]. Nonetheless, treatments’ effectiveness in CIDP should be evaluated with neurological and electrophysiological examinations and customised individually.

The treatment for CIDP focuses mainly on alleviating symptoms and restoring as much function as possible. Evidence gleaned from clinical trials and observational studies is often utilised as the foundation for developing treatment strategies for CIDP. Intravenous immunoglobulin, corticosteroids, plasmapheresis, and immunosuppressive drugs are some of the more common treatments for this condition. The particular course of treatment will be decided based on the patient’s circumstances, such as the severity of the condition and the patient’s response to treatment. It is essential to keep a close eye on the patient’s condition and make any required adjustments to the treatment at frequent intervals.

## 11. Possible Courses of Future Research to Take Regarding the CIDP

The CIDP is a multifaceted and multifactorial illness, and there is still a great deal to learn about the disease and how to treat it. Future research should concentrate on developing novel and improved treatments and gaining a deeper understanding of the underlying mechanisms responsible for CIDP. In addition, there is a demand for both broader and longer-scope studies to evaluate the efficacy of various treatments and deepen our understanding of the connection between CIDP and other neurological diseases.

## 12. The Influence of Chronic Infectious Disease on One’s Functioning and Quality of Life

CIDP might significantly impact a patient’s quality of life and ability to function. The symptoms of CIDP can be very debilitating, as they influence a person’s ability to function physically, mentally, and emotionally. It is essential to offer patients psychological support to assist them in managing the effects of the condition on their lives. The treatment for CIDP is aimed at reducing symptoms and increasing functioning, but it is also essential to do so. In addition, it is necessary to evaluate the patient’s functioning and quality of life at regular intervals throughout therapy to guarantee that the patient receives the highest level of clinically feasible care.

## 13. Conclusions

The destruction of peripheral nerves and nerve roots typifies CIDP. Moreover, it is an autoimmune disease that influences the myelinating constructions of PNS. It can be progressive, expanding over eight weeks. CIDP is typical, symmetric, involving proximal and distal sensory or motor factors, containing a focal or multi-focal distal latency prolongation. Various treatments show promising results, but treatment choices should be customised to each patient’s needs. Surveys have proved aberrations in biomarkers P100, N145, and N175 compared to healthy subjects. Patients submitted in treatment for CIDP showed better results in diagnostic biomarkers.

## Figures and Tables

**Table 1:** Symptoms and signs of typical and atypical forms of CIDP.

Typical CIDP		
	Signs	Symptoms
	Absent or reduced tendon reflexes in all limbs	Gradual or recurring, similar weakening of muscles in both the upper and lower limbs, affecting the closer and farther parts of the limbs, accompanied by sensory issues in at least two limbs
Atypical CIDP		
Variant	Signs	Symptoms
DADS	Normal or low DTR in proximal areas	Distal sensory loss and muscle weakness, predominantly in the lower limbs
Multifocal CIDP	Tendon reflexes may be normal in unaffected limbs	Loss of sensation and muscle weakness occur in multiple areas, often with uneven distribution, typically showing a preference for the upper limbs and affecting more than one limb
Focal CIDP	Low DTR	Loss of sensation and muscle weakness restricted to a single limb
Motor CIDP	Low DTR	Symptoms related to movement and physical indications without any associated sensory issues
Sensory CIDP	Absent or low DTR inall limbs	Sensory indications and signs present without any associated involvement of movement or motor functions

## Abbreviations

CIDP	Chronic Inflammatory Demyelinating Polyneuropathy
CNS	Central Nervous System
DADS	Distal Acquired Demyelinating Symmetric Neuropathy
DTR	Deep Tendon Reflex
HSCT	Hematopoietic Stem Cell Transplantation
IVIG	Intravenous Immunoglobulin
SCIG	Subcutaneous Immunoglobulin
SLE	Systemic Lupus Erythematosus
VEPs	Visual Evoked Potentials
